# Trace element associated reduction of norleucine and norvaline accumulation during oxygen limitation in a recombinant *Escherichia coli* fermentation

**DOI:** 10.1186/1475-2859-12-116

**Published:** 2013-11-21

**Authors:** Michael Biermann, Julia Linnemann, Uwe Knüpfer, Sebastian Vollstädt, Bettina Bardl, Guido Seidel, Uwe Horn

**Affiliations:** 1 Leibniz-Institute for Natural Product Research and Infection Biology (HKI), Beutenbergstrasse 11a, 07745 Jena, Germany; 2 Wacker Biotech GmbH, Hans-Knöll Strasse 3, 07745 Jena, Germany

**Keywords:** *Escherichia coli*, Norleucine, Norvaline, Trace elements, Recombinant antibody, Bioprocess design, Protein biopharmaceutical, Scale-up, Scale-down

## Abstract

**Background:**

Norleucine and norvaline belong to a group of non-canonical amino acids which are synthesized as byproducts in the branched chain amino acid metabolism of *Escherichia coli*. The earlier observed misincorporation of these rare amino acids into recombinant proteins has attracted increasing attention due to the rising use of protein based biopharmaceuticals in clinical application. Experimental data revealed pyruvate overflow inducing conditions, which typically occur in oxygen limited zones of large-scale fermentations as a major reason leading to norvaline and norleucine synthesis during *E. coli* cultivation. Previous approaches to suppress misincorporation of norleucine and norvaline considered growth media supplementation with the relevant canonical isostructural compounds, but no research was performed on the impact of the overflow metabolism related trace elements molybdenum, nickel and selenium. These elements form essential parts of the formate hydrogen lyase (FHL) metalloprotein complex, which is a key enzyme of anaerobic pyruvate metabolism in *E. coli* and could therefore represent a crucial connection to the pyruvate accumulation associated biosynthesis of rare amino acids.

**Results:**

In this study, the trace element associated response of recombinant antibody producing *E. coli* to oxygen limitation at high glucose concentration with a special focus on non-canonical amino acids was analysed. During fed-batch cultivation with provoked oxygen limitation and glucose excess norleucine and norvaline were only accumulated in the absence of molybdenum, nickel and selenium. In contrast, the trace element supplemented stress fermentation showed significantly reduced concentrations of these rare amino acids and the major signature fermentation product formate, supporting the correlation between a functional formate hydrogen lyase complex and low unspecific amino acid synthesis under oxygen limitation at high glucose concentration.

**Conclusions:**

The formation of norleucine and norvaline by recombinant *E. coli* during cultivation with provoked oxygen limitation and glucose excess can be reduced to levels at the detection limit by adding the trace elements molybdenum, selenium and nickel to the fermentation medium. Even under the metabolic burden during induction phase the physiologically available concentrations of non-canonical amino acids remained low. Since our results allow facile process changes that can be easily implemented to avoid the undesirable accumulation of norleucine and norvaline, we consider this study highly interesting for improved process development in *E. coli* based recombinant drug production and the future development of possible mechanisms to reduce misincorporation events into protein based biopharmaceuticals.

## Background

Beside the 20 canonical amino acids building up proteins in nature, the rare amino acids norleucine and norvaline can be formed by *E. coli* and some other gram-negative species [1,2]. Both belonging to a group of branched-chain amino acid analogues, norleucine was originally observed in regulatory mutant strains of *Serratia marcescens*[1], whereas norvaline was found to be part of an antifungal peptide secreted by *Bacillus subtilis*[3].

More recently non-canonical amino acids have moved into focus when their incorporation into recombinant biopharmaceuticals during expression in *E. coli* was detected. Prominent cases of therapeutically relevant proteins with confirmed misincorporation are interleukin 2 and human brain derived neurotrophic factor showing norleucine substitutions, as well as recombinant haemoglobin with norvaline sequence variation [4-8].

According to the guidelines of regulatory health authorities like the US Food and Drug Administration (FDA) and European Medicines Agency (EMA), all of this modified variants of the final drug product need intensive analytical characterization and are therefore highly undesirable in biopharmaceutical production [9,10]. Furthermore, there is only poor knowledge on the possible deleterious effects of norleucine and norvaline containing protein therapeutics in humans s, especially concerning an unwanted immunogenic response [11]. As a result of the high potential diversity of human antibody reservoir, a by-product induced autoimmune cross-reaction against endogenous proteins could lead to severe health problems in patients [12,13]. And although the phenomenon of amino acid misincorporation is not regarded as an issue of relevant ICH Q6B guidelines, it most likely becomes a particular regulatory concern due to the increasing number of corresponding reports. In addition to observed by-product formation in *E. coli*, several cases of translational errors in antibody production via mammalian Chinese hamster ovary (CHO) cells were published quite recently, raising a potentially fundamental problem throughout various expression platforms [14-16]. The molecular event of norleucine and norvaline incorporation into nascent peptide chain basically occurs via misaminoacylation of the cognate tRNA [17,18]. Following the isostructural properties of norvaline for leucine and norleucine for methionine, tRNA^leu^ and tRNA^met^ are mischarged by aminoacyl-tRNA synthetases producing corresponding substitutions in the protein sequence [19,20].

The known biosynthesis of norleucine and norvaline in *E. coli* is facilitated by the promiscuous enzymes of the (iso)-leucine biosynthetic pathway [8,21,22]. Allowing the utilization of diverse keto-acid substrates the enzymes isopropylmalate-synthase (LeuA), isopropylmalate-isomerase (LeuCD) and isopropylmalate-dehydrogenase (LeuB) of the *leu*ABCD-operon are supposed to catalyse a direct chain elongation starting from the central carbon intermediate pyruvate. The enzyme specificity of LeuA in *E. coli* has not been under investigation yet, but data are available for various substrates of the highly conserved homolog in *Salmonella typhimurium* which possesses condensation activity for pyruvate with acetyl-CoA [23]. The first extension product 2-ketobutyrate acts as a crucial branched-chain amino acid precursor and is synthesized from threonine under standard conditions. In the subsequent reaction course 2-ketobutyrate is further elongated towards the intermediates 2-ketovalerate and 2-ketocaproate which are finally transaminated to norleucine and norvaline by the aminotransferases IlvE, TyrA and AvtA.

Despite these facts, there exists just a limited understanding of the metabolic conditions leading to the synthesis of non-canonical amino acids in *E. coli*. Current physiological studies revealed strong evidence for the connection of glucose overflow metabolism and pyruvate accumulation to the occurrence of norvaline in wild-type *E. coli* fermentations [22,24]. This is further supported by knock-out experiments showing that deletion of the *ilvA* gene, which is responsible for 2-ketobutyrate synthesis from threonine, results in significant higher cellular norleucine and norvaline concentrations [20].

Since the effect of amino acid misincorporation is of main interest in the area of industrial production of biopharmaceuticals, the analysis of recombinant large-scale overflow metabolism related to norleucine and norvaline formation by *E. coli* is an essential prerequisite for the development of any based process to avoid mistranslation. Furthermore, *E. coli* represents the major bacterial workhorse for the production of protein therapeutics, currently providing 30% of all FDA and EMA approved recombinant drugs [24].

Numerous studies describe the metabolic impact of culture heterogeneities as a result of imperfect mixing in large-scale bioprocesses [25,26], but surprisingly no studies are accomplished on stress induced formation of rare amino acids in recombinant *E. coli* cultivations related to anaerobic overflow metabolism. With this in mind, our current research was inspired by a report from Soini et al., which discusses the positive influence of additional metal trace elements on anaerobic overflow reactions in *E. coli* scale-down experiments [27]. The authors showed that media supplementation with selenium, nickel and molybdenum prevents toxic formate accumulation under glucose excess and oxygen limited conditions through a fully functional formate hydrogen lyase enzyme (FHL) complex. However, the experiments of Soini et al. were carried out using the *E. coli* wild-type strain W3110 without including the additional metabolic burden imposed on cell physiology by synthesizing a heterologous protein. We therefore decided to employ the expression of the well characterized synthetic antibody domain B10 in *E. coli* RV308 as a relevant example for protein based biopharmaceuticals under current clinical interest [28]. In three representative fed-batch cultivation set-ups the impact of molybdenum, nickel and selenium on norleucine and norvaline accumulation under oxygen limitation and glucose excess provoking conditions was analysed and compared to a control fermentation with sufficient oxygen supply. With this approach we want to further broaden the understanding of norleucine and norvaline accumulation in recombinant *E. coli* bioprocesses.

In this study we show the reduction of free norleucine and norvaline formation under oxygen limited stress environment by medium supplementation with the FHL-related trace elements. We therefore hypothesize that potential channelling of the pyruvate knot towards formate disproportion may feature a lower production of non-canonical amino acids under large-scale production conditions. Here we report for the first time a trace element associated approach for the reduction of physiologically available unwanted amino acid species in recombinant *E. coli* fermentation processes.

## Results and discussion

### Dynamics of growth behaviour and glucose utilization during oxygen downshift experiments

Since the metabolic response towards basic environmental stress factors such as oscillating oxygen or substrate supply may result in reduced productivity [26,29-31] or causing severe modifications of the desired protein product, a deeper understanding of fundamental mechanisms in relevant biopharmaceutical processes is highly desirable.

**Figure 1:**
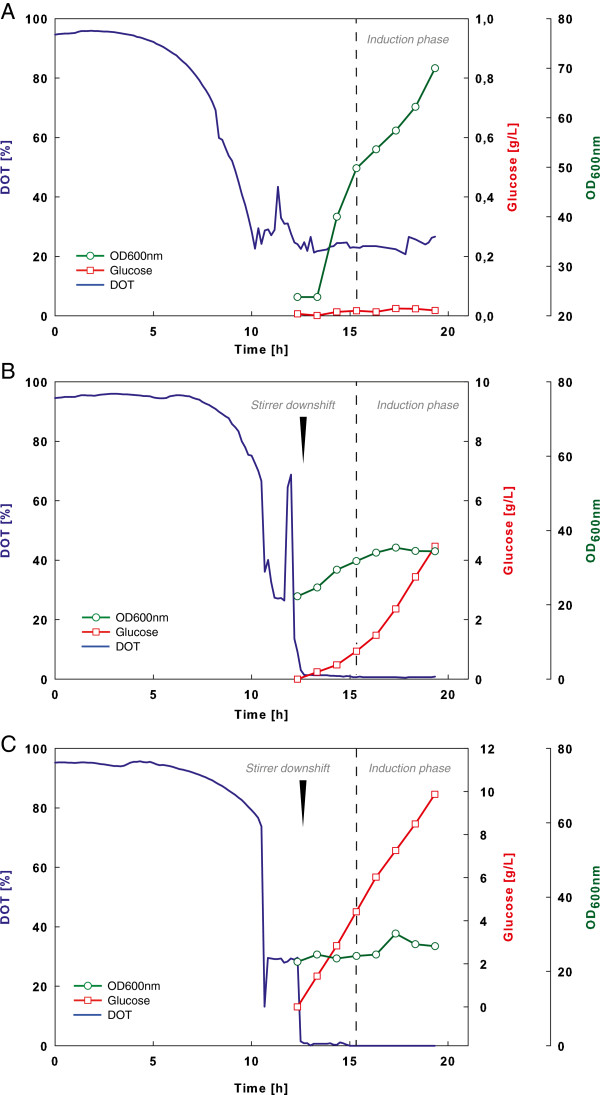
***E. coli *RV308 growth dynamics during different cultivation modes used in this study.** The graphs illustrate experimental data for optical density (OD_600nm_), DOT and glucose concentration. **(A)** Glucose limited fed-batch cultivation on mineral salt medium with continuous feed of glucose at constant feed rate of 10 g L^-1^ and indicated time point of induction (dashed line). **(B)** and **(C)** same as **(A)** but with a downshift of DOT by a decrease of stirrer speed (arrow) and either without **(B)** or with **(C)** the addition of extra trace elements to the fermentation medium. Stirrer downshift in **(B)** and **(C)** was performed 20 min before the start of glucose feeding to ensure glucose excess during cultivations. Samples were collected from the beginning of continuous glucose feed.

In consequence the aim of this work focussed on the so far disregarded influence of supplementary molybdenum, nickel and selenium on the formation of the rare amino acids norleucine and norvaline during an oxygen downshift in recombinant *E. coli*. We therefore chose an *E. coli* RV308 production strain, expressing the camelid antibody domain B10 as a model for possible target molecules underlying current clinical interest. In order to compare the obtained results to balanced cultivation conditions a glucose limited fed-batch reference set-up with dissolved oxygen tension (DOT) above 20% was performed (see Figure 1A). To ensure large-scale stress conditions, oxygen limitation and glucose excess during cultivation was provoked by a stepwise stirrer down-shift and continuous linear feeding in the scale-down fermentation set-ups (see Figure 1B,C). To allow proper analysis of the *E. coli* cultivation samples, the oxygen downshift was started at optical densities of about OD_600nm_ = 35, since lower cell densities showed crucially varying results in amino acid analysis (unpublished data). The growth of *E. coli* during reference fermentation indicated typical substrate consumption and increasing biomass production up to a high cell density of OD_600nm_ = 72 at the end of induction phase. In the experiments wherecells were exposed to an permanent oxygen downshift, cell growth during induction phase arrested at lower levels around OD_600nm_ = 30 as expected. The oxygen limited cultivation with additional Mo, Ni and Se showed slightly lower cell densities between OD_600nm_ = 26 and 32 during the induction phase compared to the cultivation without extra trace elements with an OD_600nm_ between 30 and 36. This effect is very likely a consequence of an increased carbon loss through a higher carbon dioxide production related to active FHL or an increased lactate accumulation which was observed in similar experiments with permanent oxygen downshift [27]. Concerning the lowered biomass yield as a critical concern in industrial protein production, the addition of Mo, Ni and Se could still be an option to test, since the authors also reported no negative effects during experiments with oscillating oxygen and glucose concentrations.

In addition, during the oxygen limited cultivation a glucose accumulation of 4 g L^-1^ glucose without and up to 10 g L^-1^ glucose with addition of extra trace elements at the end of induction time was observed. These results clearly correspond to earlier studies, showing a strong inhibition on growth and glucose consumption when *E. coli* is impaired with both, metabolic burden of recombinant expression and overflow metabolism [27,32,33].

### Reduction of norleucine and norvaline accumulation by medium supplementation with molybdenum, nickel and selenium

**Figure 2:**
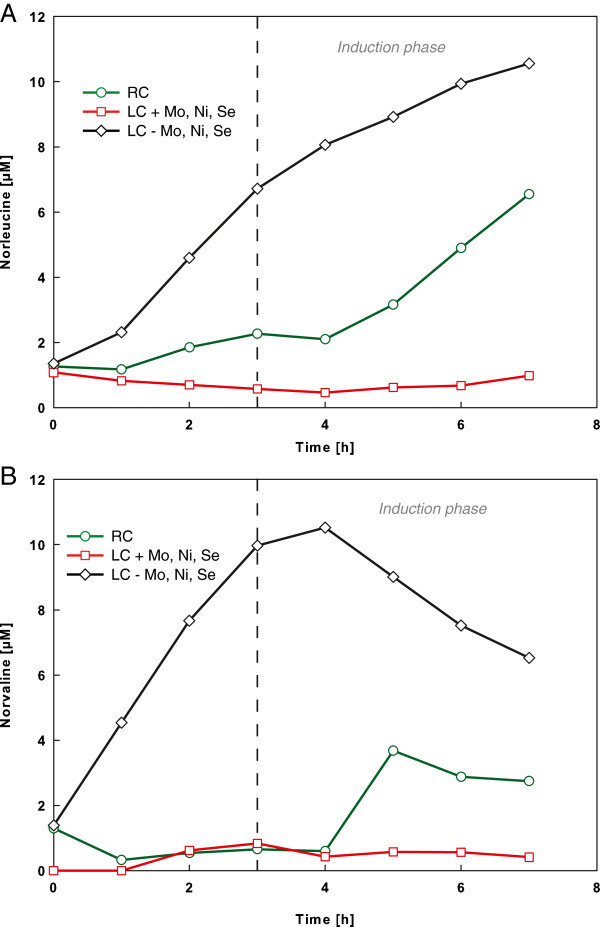
**Analysis of non-canonical amino acids from recombinant *E. coli *RV308 fed-batch cultivations.** Concentration time course of norleucine **(A)** and norvaline **(B)** during cultivation without (square) and with oxygen limiting conditions (circle and quadrate) from the time point of feed start (zero hours). Dashed vertical lines indicate the process times for start of recombinant expression of B10.

The single most striking observation to emerge from the data comparison was the significant reduction of norleucine and norvaline accumulation by medium supplementation with molybdenum, nickel and selenium (Figure 2A and B). In the oxygen limited cultivation set-up with additional trace elements surprisingly low concentrations of 0.57 μM norleucine and 0.83 μM norvaline were detected before induction and remained below that until the end of the cultivation. In contrast, norleucine and norvaline accumulated up to 6.72 μM and 9.97 μM in the fermentation set-up without the addition of Mo, Ni and Se only 3 h after feed start and further increased during the expression of B10. The time course analysis of the reference cultivation revealed an increase of the non-canonical amino acids upon induction of the recombinant expression with isopropyl-β-D-thiogalactopyranosid (IPTG) (Figure 2). Since the concentrations of norleucine and norvaline showed the highest accumulation rates upon the induced oxygen limitation and a substantial decrease before the time point of induction, the relevance of oxygen limitation and glucose excess as the main cause of unspecific biosynthesis of these non-canonical amino acids is futher supported. By a simply addition of Mo, Ni, and Se to the cultivation medium the accumulation of physiological available unwanted non-canonical amino acids was significantly reduced, which can become particularly relevant to protein-based biopharmaceutical production processes[11,16]. However, further investigations are essential to assess the effect of reduced free norleucine and norvaline concentrations on misincorporation rates in amino acid sequence of the final protein product.

### Differences in formate accumulation and recombinant expression of antibody domain B10

**Figure 3:**
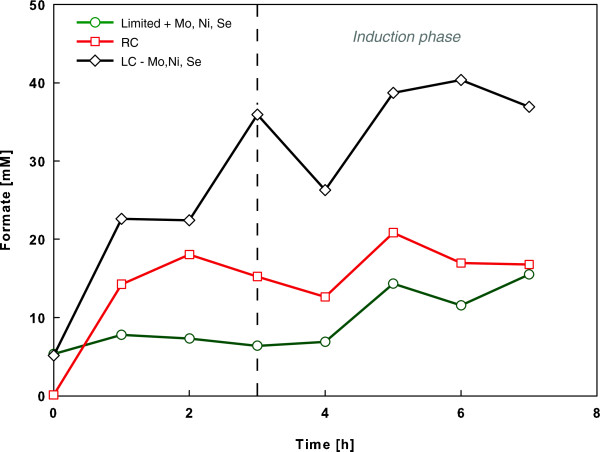
**Formate accumulation profiles in the performed cultivation types of *E. coli *RV308 with recombinant expression of B10.** The graphs show data from cultivations without (square) and with oxygen limiting conditions (circle and quadrate) from the time of feed start (zero hours) and induction phase (vertical dashed line indicates time point of induction).

The time course analysis of formate concentration during the different *E. coli* cultivation set-ups indicated a reducing effect to formate accumulation by additional Mo, Ni and Se in the mineral salt medium in the oxygen limited culture. An overall 2-fold increased formate concentration of up to 40 mM was measured in the oxygen limited fermentation without additional trace elements (Figure 3). In the large-scale mimicking cultivation performed with Mo, Ni and Se supplemented medium, the extracellular formate concentration was more than 50% lower at the end of the induction phase compared to the standard medium set-up. The obtained data of formate accumulation were consistent with previous studies showing the beneficial effect of additional Mo, Ni and Se in the mineral salt cultivation medium of anaerobic *E. coli* cultivations [27].

Besides this, the correlation between a higher cellular capacity to disproportionate formate by cultivation in supplemented medium and a higher recombinant expression level of B10 was apparent (Table 1). A final B10 protein yield of 72.6 mg/L (without additional Mo, Ni, Se) and 137.5 mg/L (with additional Mo, Ni, Se) was measured for the oxygen limited cultivations, whereas the reference fermentation conditions ensuring sufficient oxygen supply, showed an approximately 10-fold higher antibody yield of 2056.2 mg/L (Table 1). The latter observation of high recombinant expression is not surprising, since the central carbon metabolism of the recombinant *E. coli* cells was ideally balanced by optimal glucose feeding rate and oxygen supply, keeping the metabolic burden as low as possible [32,33].

**Table 1:** **Analysis of recombinant protein yield, OD**_**600nm **_**at end of cultivation and stirrer rate during expression phase of *E. coli *RV308 B10**

**Cultivation type**	**Maximum stirrer speed (RPM)**	**OD**_**600nm**_	**Protein yield (mg/L)**
Reference	1000	73	2056.2
Oxygen downshift	500	35	72.6
Oxygen downshift + Mo, Ni, Se	500	31	137.5

The protein yield data are given as the mean between three measurements.

Connecting these findings with significantly lowered norleucine and norvaline accumulation in the respective fermentation scenarios, one might hypothesize that a functional FHL enzyme complex can channel the pyruvate flux to disproportion of formate and avoiding accumulation of unspecific precursors for non-canonical amino acids [24,34].

## Conclusions

In this study we could clearly show the trace element associated reduction of norleucine and norvaline accumulation during oxygen downshift experiments of biopharmaceutical relevant recombinant *E. coli* fermentations. We identified the elements molybdenum, nickel and selenium as key cultivation medium components reducing the formation of non-canonical amino acids under oxygen limitation and glucose excess. Our findings might contribute to the development of new strategies to avoid norleucine and norvaline misincorporation into protein based drugs and further improve recombinant pharmaceutical production in accordance to current international guidelines.

## Methods

### Strain

The strain used in this study was *Escherichia coli* K-12 RV308 ATCC 31608x [su^-^, ΔlacX74, gal IS II::OP308, strA] transformed with plasmid p41B10aP [28]. Stock solution of the strain was stored in 25% [v/v] glycerol solution supplemented with mineral salt medium.

### Precultures

All precultures were cultivated at 30°C on a rotary shaker set to 200 rpm. The initial precultures were performed in 10 mL of LB medium supplemented with ampicillin (100 μg mL^-1^) in 100 mL Erlenmeyer flasks for 8–12 hours inoculated with *E. coli* stock solution. The second precultures for inoculation of bioreactors were performed in 1000 mL Erlenmeyer flasks filled with 100 mL of the respective bioreactor mineral salt medium supplemented with 10 g L^-1^ glucose.

### Bioreactor cultivations

The fed-batch cultivations were performed in a Sixfors bioreactor (Infors, Switzerland) equipped with DCU-3 controlling unit and MFCS-monitoring system (Sartorius, Germany) starting with 0.4 L working volume. Main cultures were inoculated with liquid precultures to a starting OD_600_ of 0.15. The defined mineral salt cultivation medium was prepared in distilled water containing per L: 8.6 g Na_2_HPO_4_ × 12 H_2_O, 0.5 g NaCl, 3 g KH_2_PO_4_, 1 g NH_4_Cl, 10 mL Fe-citrate (0.023 M), 0.1 mL EDTA (0.2 M), 0.1 mL CoCl_2_ × 6 H_2_O (0.1 M), 0.1 mL MnCl_2_ × 4 H_2_O (0.75 M), 0.1 mL CuCl_2_ × 4 H_2_O (0.1 M), 0.1 mL H_3_BO_3_ (0.5 M), 2 mL Zn(CH_3_COO)_2_ × 2 H_2_O (0.018 M), 5 mL MgSO_4_ (1 M), 0.1 g thiamine hydrochlorid and 1 mL antifoam agent. In order to examine the effect of medium supplementation with molybdenum, nickel and selenium, these additional trace elements were initially added to the indicated cultivation to a final concentration of: 0.24 mg L^-1^ Na_2_Mo × 5 H_2_O, 1.45 mg L^-1^ Ni(NO_3_)_2_ × 6 H_2_O and 0.17 mg L^-1^ Na_2_SeO_3_ × 5 H_2_O [27]. The cultivation temperature was 30°C and pH was kept at 6.8 by controlled feeding of 10% ammoniumhydroxid. The fermentation media contained an initial glucose concentration of 15 g L^-1^, the feed solution contained 500 g L^-1^ glucose. Reference cultivation was performed in glucose limited fed-batch mode with controlled DOT set point at 30%. The metabolic shift in scale-down fermentations was initialized at the end of exponential growth phase by lowering the stirrer rate from 1000 rpm to 500 rpm. The glucose feeding was started 20 min after stirrer downshift with a constant rate of 10 g L^-1^ h^-1^ to ensure glucose excess during the whole cultivation. For protein expression, cultures were induced with 1 mM IPTG.

### Analysis of cell growth

Cell growth was monitored by measurement of absorbance (OD_600nm_) with a Spekol spectrophotometer (Carl Zeiss, Germany).

### Amino acid analysis

Fermentation samples for amino acid analysis of clarified crude broth lysates were immediately quenched in −40°C cold 60% methanol and subsequently shock-frozen in liquid nitrogen. Samples were stored at −80°C until analysis. The sample preparation for HPLC included dilution of samples to the same cell density with 0.9% NaCl, a subsequent sonification step for 10 min on ice, removal of cell debris and deproteinization by centrifugation for 10 min (4°C, 16.000 × g) and ultracentrifugation of supernatant using Amicon Ultra 3 kDa cut-filter devices (Millipore. The HPLC analysis was performed with a JASCO X-LC HPLC system (JASCO Corporation, Japan), containing autosampler unit, intelligent column thermostat, fluorescence detector and a Nucleodur C18 Gravity column (Macherey-Nagel, Germany). Pre-column derivatization of 30 μL sample solution and 30 μL OPA reagent (Sigma-Aldrich, Germany) was carried out for 30 s in autosampler unit, followed by injection of 1 μL onto column. HPLC analysis was further performed as described earlier [34].

### Analysis of glucose and formate

Glucose and formate concentrations were analysed from medium samples, which were immediately centrifuged for 5 min (4°C, 16.000 × g), filtered (0.2 μm) and shock-frozen in liquid nitrogen. Glucose was analysed with ECA 2000 unit (YSI Life Sciences, USA). Formate was analysed with Formiat Assay Kit (Bio Vision, Germany).

### Quantification of B10aP

Determination of correctly folded B10aP was performed by enzyme activity assay and calculated by means of a standard curve. Colorimetric detection was performed with the PNPP (p-nitrophenyl phosphate, disodium salt) substrate (Pierce) for alkaline phosphatase. Samples of 1 mL were taken before and every hour during induction phase, followed by immediate shock-freezing in liquid nitrogen, sonification for 10 min on ice and removal of cell debris by centrifugation.

## Competing interests

The authors declare that they have no competing interests.

## Authors’ contributions

MB carried out the experiments and drafted the manuscript. JL and UK participated in the fermentation experiments and sample preparation. SB and BB performed HPLC analysis. UH and GS conceived of the study, and participated in its design and coordination and helped to draft the manuscript. All authors read and approved the final manuscript.
